# Clinical Utility and Functionality of an Artificial Intelligence–Based App to Predict Mortality in COVID-19: Mixed Methods Analysis

**DOI:** 10.2196/27992

**Published:** 2021-07-28

**Authors:** Ahmed Abdulaal, Aatish Patel, Ahmed Al-Hindawi, Esmita Charani, Saleh A Alqahtani, Gary W Davies, Nabeela Mughal, Luke Stephen Prockter Moore

**Affiliations:** 1 Chelsea and Westminster NHS Foundation Trust London United Kingdom; 2 National Institute for Health Research Health Protection Research Unit in Healthcare Associated Infections and Antimicrobial Resistance Imperial College London London United Kingdom; 3 Johns Hopkins University Baltimore, MD United States; 4 King Faisal Specialist Hospital and Research Centre Riyadh Saudi Arabia; 5 North West London Pathology Imperial College Healthcare NHS Trust London United Kingdom

**Keywords:** app, artificial intelligence, coronavirus, COVID-19, development, function, graphical user interface, machine learning, model, mortality, neural network, prediction, usability, utility

## Abstract

**Background:**

The artificial neural network (ANN) is an increasingly important tool in the context of solving complex medical classification problems. However, one of the principal challenges in leveraging artificial intelligence technology in the health care setting has been the relative inability to translate models into clinician workflow.

**Objective:**

Here we demonstrate the development of a COVID-19 outcome prediction app that utilizes an ANN and assesses its usability in the clinical setting.

**Methods:**

Usability assessment was conducted using the app, followed by a semistructured end-user interview. Usability was specified by effectiveness, efficiency, and satisfaction measures. These data were reported with descriptive statistics. The end-user interview data were analyzed using the thematic framework method, which allowed for the development of themes from the interview narratives. In total, 31 National Health Service physicians at a West London teaching hospital, including foundation physicians, senior house officers, registrars, and consultants, were included in this study.

**Results:**

All participants were able to complete the assessment, with a mean time to complete separate patient vignettes of 59.35 (SD 10.35) seconds. The mean system usability scale score was 91.94 (SD 8.54), which corresponds to a qualitative rating of “excellent.” The clinicians found the app intuitive and easy to use, with the majority describing its predictions as a useful adjunct to their clinical practice. The main concern was related to the use of the app in isolation rather than in conjunction with other clinical parameters. However, most clinicians speculated that the app could positively reinforce or validate their clinical decision-making.

**Conclusions:**

Translating artificial intelligence technologies into the clinical setting remains an important but challenging task. We demonstrate the effectiveness, efficiency, and system usability of a web-based app designed to predict the outcomes of patients with COVID-19 from an ANN.

## Introduction

Clinical big data that are being collated in many health care settings have enabled prognostic scores to be developed on the basis of classical regression analysis, but these models frequently rely on laboratory parameters (which are not available in many primary care settings and in some low- and middle-income settings) [1]. Furthermore, because of a priori assumptions, these regression models may fail to leverage the data fully to create accurate prognostic models. Artificial intelligence (AI) techniques represent a potential solution [2], allowing more comprehensive use of big data, including the potential identification of proxy indicators (such as symptomatology and comorbidities) for laboratory parameters that may predict COVID-19 outcomes. Such systems have been shown to be accurate and reliable when compared to traditional regression models [3,4]. However, one of the principal challenges in leveraging AI clinically for COVID-19 has been in translating systems to the clinical setting [5].

Developing systems to accurately predict COVID-19 outcomes has several potential benefits at the patient, departmental, and organizational levels. At the patient level, predictive models would allow for early critical care reviews of high-risk patients and early discussions regarding treatment escalation plans. Medical departments could estimate bed requirements and account for intensive care unit (ICU) resource allocation issues more accurately. In turn, health care organizations could better manage staffing levels and health care resource procurement and distribution.

We describe here the clinical operationalization of an artificial neural network (ANN) that produces patient-specific mortality predictions for patients with COVID-19 [3,4] and explore the development of a graphical user interface (GUI) to facilitate the use of the system at the bedside. Subsequently, we assessed the utility and functionality (measuring effectiveness, efficiency, and satisfaction) of the GUI, which leverages this ANN, and analyzed the translational pathway for its integration and use in a clinical setting.

## Methods

### Development of the ANN

An ANN was developed, as previously described [3,4], to prognosticate for patients with COVID-19. A multilayer perceptron was trained and validated with 398 patients from a single London hospital, with an input of 22 features selected in accordance with previous studies [6-8], in turn developed after a review of existing evidence of contributory factors [9,10]. Demographics included gender and age. Smoking history was also included. Comorbidities included the presence or absence of asthma, chronic obstructive pulmonary disease, or chronic respiratory disease; hypertension; diabetes; congestive cardiac failure; ischemic heart disease; chronic kidney disease; hepatic cirrhosis; or a history of cerebrovascular events. Symptom data included the presence of absence of fever, cough, dyspnea, myalgia, abdominal pain, diarrhea, vomiting, altered mentation, collapse, and olfactory change or ageusia, as well as the duration of symptoms prior to hospital admission. Data were anonymized at the point of extraction and encoded from patient electronic health records by 3 health care practitioners (EC, AP, and A Abdulaal).

The model weights were initialized with Xavier normal initialization, and a dropout of 20% and 40% were used on the 2 hidden layers. Euclidean (L2) regularization was further added to the hidden layers to further prevent overfitting. The model was trained with 318 patients, and model hyperparameters were optimized on the basis of 10-fold cross-validation of the training set. The ANN was then trained on the full training set and validated on a held-out test set of 80 patients. For each patient input, the model produces a single output by using a sigmoid activation function (which demarcates results between 0 and 1). This output represents the probability of death during the current hospital admission for the patient. Discriminative ability was measured using the area under the receiver operating characteristic curve, and calibration was assessed both visually and by using the Brier score.

Data were collected as part of routine care by the responsible clinical team. No patient-identifiable data were used in this analysis. The study protocol was approved by the antimicrobial stewardship group at Chelsea & Westminster NHS Foundation Trust. The need for written informed consent was waived by the Research Governance Office of Chelsea & Westminster NHS Foundation Trust. The study was conducted in accordance with the tenets of the Helsinki declaration.

### Development of GUI

A web-based app was developed using Node.js, an open-source, cross-platform, javascript runtime environment [11]. Express [12], a web-based framework for Node.js, which provides a set of tools for app development, was used to build the backend of the app. A combination of Nielsen and Shneiderman heuristics of user interface design were used to generate the initial GUI [13]. An iterative development process based on usability assessments throughout the design cycle was used to develop the interface further, thus ensuring its intuitiveness and ease of use. The app is currently developed as an English-language app.

**Figure 1:**
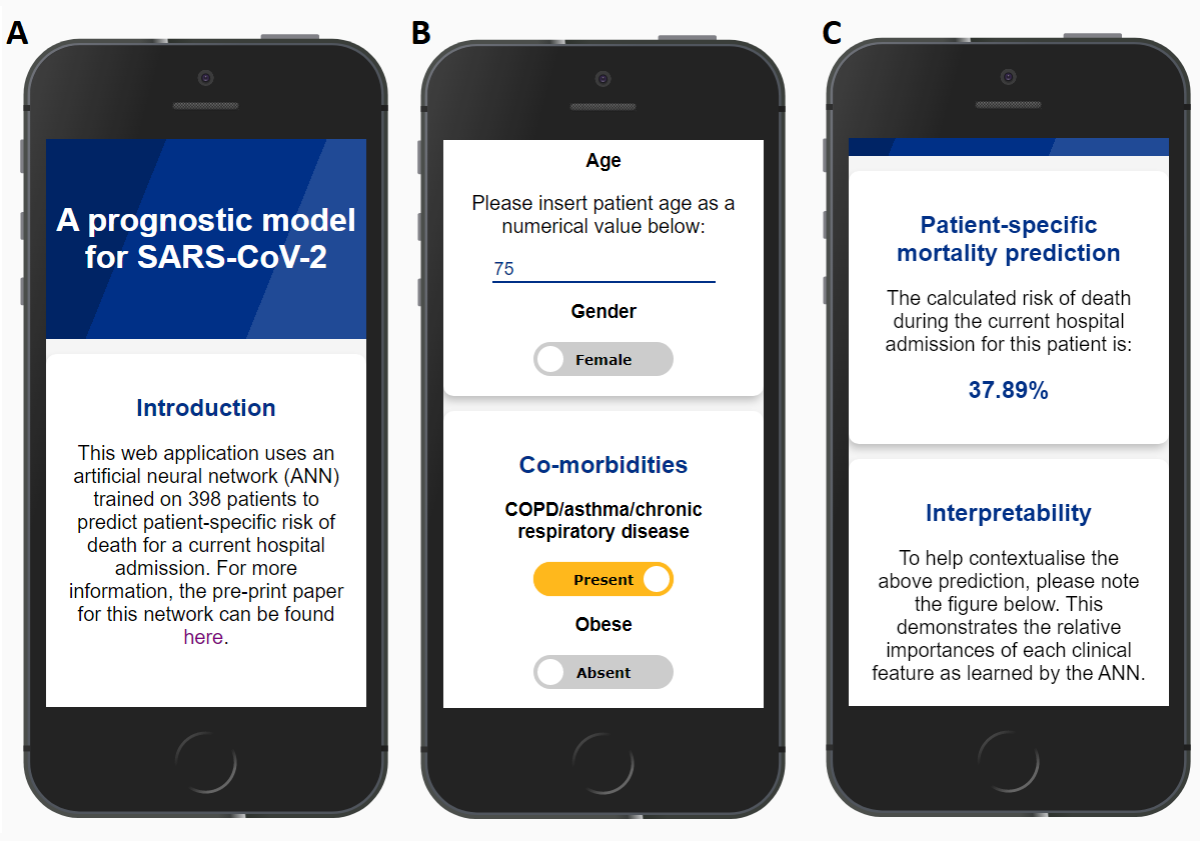
Screenshots of the initial artificial neural network (ANN)–based COVID-19 prognostication app. (A) The introductory screen with a hyperlink to access more data on the ANN and its development. (B) The data input process with examples of numerical and categorical features. Selected categorical features are color-coded and labeled. Numerical features have input instructions above the data collection field. (C) A portion of the results screen. Patient mortality data are presented as a human-readable percentage.

The app collects patient demographics, comorbidities, and symptomatology data [4]. The data are then converted into a normalized tensor (a multidimensional array of data, which can be read by a machine learning algorithm [14]) in the browser. On the backend, these data are fed into the ANN [4] (the deep learning library Tensorflow.js [15] was used to transfer the data to the Node.js server), which makes a patient-specific mortality prediction, and the result is then returned to the user (Figure 1). The relative importance of patient-level factors with respect to the mortality prediction are displayed as a static figure on the results page. No patient data are stored by the app after a prediction is made, and the app can be used for a new patient by navigating to the home screen.

### Study Design

This was a between-subjects study with 1 condition: all participants used the app to predict the mortality risk for several patients. Effectiveness was defined as successful completion of a task. This was measured by assessing whether participants were able to insert a complete patient data set into the app GUI and successfully navigate to the results screen. Efficiency was defined as the duration to complete a task, which was measured by timing participants for each patient-specific data set that they inserted into the app. This time period was measured from when participants finished reading the introductory paragraphs until successful navigation to the results screen. Satisfaction was defined as a participant’s perception of the effectiveness and efficiency of the app. Satisfaction was measured using the System Usability Scale (SUS) [16]. A semistructured interview format was used after the SUS assessment to gather additional feedback on the app. This allowed for flexible data collection with open-ended responses while ensuring that relevant topics were covered [17,18].

### Participants

Several key informants [19] were selected across different clinical settings and seniority levels to represent the varied roles in managing patients with COVID-19. For example, initial assessment of the patient might be carried out by a junior physician in the emergency department, while a senior physician could be involved in critical decision-making, such as the establishment of treatment escalation plans.

Data saturation, defined as the point at which additional data would not add new information or require changes to be made to the developed findings, was estimated to occur at 30-35 interviews [20]. Participants were recruited in person at a single hospital site. We used maximum variation and snowball sampling to increase the likelihood that findings represent a wide range of perspectives with regard to the semistructured interviews [18,21].

### Materials and Procedure

Informed written consent was obtained from all participants. Participants were made aware of their right to withdraw from the study at any point during data collection. Data were anonymized for all participants, except for designation and age because these data were considered important for contextualizing findings.

Demographic data and experience with electronics were recorded verbally, including baseline computer and smartphone app experience scores (on a scale of 1 representing novice experience, to 10 representing expert experience). Three fictitious patient data sets in the form of clerking sheets (medical histories) were provided to each participant. Participants then entered the data into the app to generate a patient-specific mortality prediction on a computer device. This section of the assessment was timed.

While participants used the app, effectiveness and efficiency measures were collected. Once the tasks were completed, participants were provided with the SUS assessment on a web-based survey data collection platform, and the semistructured interview was then conducted. Audio recordings of the interviews were stored on a mobile device and transcribed using Otter.ai and then analyzed.

### Ethical Approval and Consent to Participate

Data were collected, as part of service development work, by the responsible clinical team. Data were anonymized at the point of extraction by the care team. The analysis protocol was approved by the Antimicrobial Stewardship Group at Chelsea & Westminster NHS Foundation Trust and this was confirmed as a service development.

### Data Analysis

Usability, as measured by effectiveness, efficiency, and satisfaction, was reported with descriptive statistics. Interview data were analyzed with a thematic framework method (by A Al-Hindawi, AP, and EC), which allowed for the development of themes from the interview narratives [22].

### Availability of data and materials

The data sets analyzed during the current study and further details on gaining access to the intervention reported within this study are available from author AA on reasonable request, as long as the local ethics and research governance criteria are met. The app is currently available in the alpha version [23].

## Results

### Results Overview

In total, 31 health care workers were recruited from a single West London teaching hospital between June and August 2020; these included 5 (16.13%) foundation physicians (year 1-2 postgraduate), 5 (16.13%) senior house officers (years 3-4 postgraduate), 15 (48.39%) registrars or equivalent (year 5-10 postgraduate), 5 (16.13%) consultants (approximately >10 years postgraduate), and 1 (3.2%) primary care general practitioner (GP). None of them were excluded from the data analysis owing to equipment failure or withdrawal from the study. Of them, 12 (38.71%) participants were female. The mean participant age was 33.06 (SD 5.59) years. The mean baseline computer experience score was 7.71 (SD 2.07), and the mean baseline smartphone experience score was 8.58 (SD 1.70).

### Effectiveness

All participants were able to complete the task. In total, 78 of 93 (83.9%) vignettes (3 vignettes provided to each participant) were completed correctly, which yielded the expected prediction results by the algorithm. The failure of participants to enter clinical parameters correctly into the GUI in 15 (16.1%) encounters was explored in the qualitative analysis explained below.

### Efficiency

**Figure 2:**
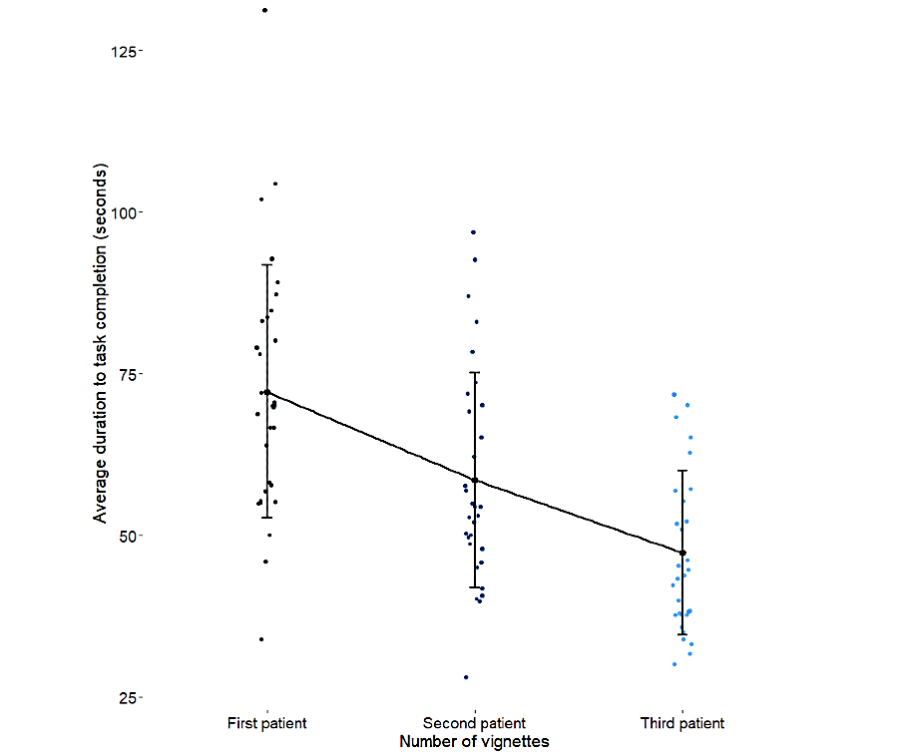
Efficiency of clinicians in using the artificial neural network–based COVID-19 prognostication app.

The mean time to complete each vignette was 59.35 (SD 10.35) seconds. Figure 2 shows the average duration of task completion for each patient vignette; participants completed the task more rapidly with each sequential attempt.

### Satisfaction

The mean SUS assessment score was 91.94 (SD 8.54). This corresponds to a grade of “A” on the University Grading Scale that was used to help interpret SUS scores [16]. This score also corresponds to an adjective rating of “excellent” on the adjective rating scale [24].

### Thematic Analysis of Semistructured Interviews

#### Uncertainty Over COVID-19 Prognostication Underpin Clinician Concerns

Regarding the management of patients with COVID-19, the physicians interviewed expressed a range of clinical concerns. Most concerns were about patient care, with the majority “worried about the deterioration of patients and their treatment escalation plan” [Participant #9, foundation physician]. Frontline physicians found themselves asking “is this the correct setting for the patient?” and “found [themselves] predicting where to manage patients” [Participant #4, consultant]. This highlighted a difference in focus depending on specialty. Physicians working in the emergency department or community were more focused on whether the patients “needed hospital admission” [Participant #18, registrar] or if they could “be managed at home” [Participant #31, GP]. In contrast, intensive care physicians were focused on “the mode of oxygen delivery needed” [Participant #25, foundation physician] and “which patients were likely to need intubation” [Participant #20, senior house officer].

In a group of physicians, there was uncertainty regarding communicating of prognoses with patients and their relatives: “Communicating that risk to the family and to the patient themselves is my biggest concern” [Participant #30, registrar].

Several physicians highlighted the fact that there was “a large amount of uncertainty in management and unpredictability in patient outcomes” [Participant #7, registrar] among patients with COVID-19. This was thought to arise from the fact that “current knowledge [of COVID-19] was poorly understood” [Participant #31, GP], and that this made “risk stratification in an unknown disease extremely difficult” [Participant #23, senior house officer].

Along with concerns about the general care of the patient and being in the appropriate care setting, there were some more specific questions that the physicians had regarding “renal, thromboembolic, and cardiac events secondary to COVID-19” [Participant #27, consultant].

#### Experience With the ANN-Based COVID-19 Prognostication App

Most physicians provided positive feedback, commenting that the app was “very well designed” [Participant #3, registrar], and “easy to pick up given I had never seen it before” [Participant #16, registrar] and that “the [GUI] is very intuitive” [Participant #1, registrar]. Many found it simple to navigate the GUI and input patient data, with the app being “not too wordy, easy to use” [Participant #9, senior house officer]. One participant liked that the application did not “need biochemical parameters,” which rendered it more “useful in [the] ED setting” [Participant #22, foundation physician], as it negated the need to wait for the results of blood tests and allowed for more rapid quantification of the patient’s risk. One clinician commented that the application allows you to “cut through noise” [Participant #24, senior house officer] when faced with a complicated case and helped to “pull different aspects together.” The result was useful, as it was “nice to have numbers that are patient-specific” [Participant #24, senior house officer].

#### Interpretation of the Predictions of the ANN-Based COVID-19 Prognostication App

Mortality risk predictions for the different vignettes elicited a range of reactions from participants. In total, 29% of physicians felt surprised by the app’s predictions. “I was surprised by how high the first mortality prediction was” [Participant #16, senior house officer]. Some clinicians felt that the app's mortality risk prediction was lower than they clinically expected. “I was surprised by some of the results, one lower than I thought” [Participant #2, registrar].

Other participants felt that the scores reflected their experience with patients with COVID-19: “Those numbers were relatively reasonable to what I have seen” [Participant #10, registrar]. One participant commented that “despite 2 of the scenarios appearing fairly similar, they had significantly different mortality predictions” [Participant #31, GP]. Overall, 6 participants felt that the mortality predictions were higher than expected, while 1 physician speculated that the app’s predictions were lower than expected. Four physicians felt that the predictions were closely aligned to their clinical judgement.

#### Impact of the ANN-Based COVID-19 Prognostication App on Clinical Practice

In cases with a clear prognosis according to the clinician, the app positively reinforced clinical decision-making. Some physicians noted that “in clinical practice, it’s quite obvious who’s going to go off” [Participant #3, registrar]. Nonetheless, some underscored the potential benefit of concordance between their clinical decision-making and the app’s predictions:

If I was planning to admit someone to ICU, this app might be useful in helping me make that decision. I’d base my management on my clinical judgement, but this might be a useful adjunct.

Participant #6, consultant

Other participants felt that the app provided them a sense of positive reinforcement:

I think it gives reassurance regarding your clinical judgement, especially if the app is roughly in agreement with your inclination.

Participant #7, registrar

Several critical care physicians focused on integrating [the score] into their own clinical judgement, and if the tool then validates [their] suspicion, it gives [them] a good positive predictive value.

Participant #17, registrar

With strong disparities, most physicians commented that they would revisit the case:

It would help you take a step back and look at the patient again irrespective of the score; I think that’s the main use of predictive calculators to me.

Participant #13, registrar

Many participants explained that when they strongly disagreed with the algorithm, they would base their management on their personal clinical judgement:

If I looked at the tool and it said to me ‘okay, she’s got a 4% chance of mortality’, but I look at the patient at the end of the bed and they appear incredibly frail, in that instance my judgement would overrule the application’s prediction.

Participant #17, registrar

When a case was speculated to be borderline, the app helped as an “adjunct to the doctor” [Participant #25, registrar], to aid in forming a general impression of the case. Furthermore, some participants felt that the app could actively “help with clinical decision-making in more complicated or borderline patients” [Participant #23, senior house officer].

Several physicians commented that the app would act as an additional tool in their decision-making process, thereby complementing their clinical judgement. In total, 14 physicians explained that the app’s results may help them stratify the risk to their patients more effectively, thus ensuring the right care setting. For example, one physician indicated that “It would allow me to risk stratify patients who are coming in; I might contact ICU earlier on” [Participant #16, registrar], and “it would be good as a screening tool to risk-stratify patients” [Participant #19, foundation physician], and “it would help me stratify future risk in an unknown disease” [Participant #20, registrar].

Many physicians felt that the app’s predictions could be used to “better communicate patient outcomes” [Participant #24, foundation physician] to the patient and their family members, as well as “between medical colleagues” [Participant #26, registrar]. Topics that physicians felt would benefit from the app’s results included “communicating disease severity” [Participant #27, consultant] and “the need for intensive care” [Participant #30, registrar] to the patient and their relatives.

Five physicians felt that the use of this app would not impact their clinical management, and one was unsure of the utility of the app:

It’s tricky; I’m not sure whether it would alter my decision making in any appreciable way, but the numbers are interesting to see.

Participant #11, consultant

However, most agree that given COVID-19 is a “new disease, having any source of prediction would be useful for guiding management, and might help as an adjunct to decide on escalation” [Participant #8, senior house officer].

#### User-Driven Evolution of the ANN-Based COVID-19 Prognostication App

Many participants noted that it would be more intuitive to elicit symptoms before comorbidities, as this workflow more closely aligns with the clinical practice of many physicians: “I found myself scrolling down to fill in some details and then scrolling up to fill in the rest” [Participant #1, registrar]. However, other participants tended to prefer inserting comorbidity data prior to symptomatology: “The flow makes more sense for my clinical practice” [Participant #2, registrar]. Two physicians felt that there were many required variables for use of the app:

It might be easier to reduce the number of variables from 20 without reducing the model’s predictive power too dramatically. This might make it easier to use.

Participant #3, registrar

However, one participant explained that this was not an important issue as the data were easy to accrue from the initial clerking:

There are a lot of yes/no boxes relative to other medical calculators, but that was alright because they were very easy to answer; data entry is elicitable from the clinical history.

Participant #4, consultant

One physician expressed being unable to find a disclaimer to explain that the app should only be used for patients with confirmed COVID-19. Similarly, 1 physician suggested the inclusion of a “disclaimer regarding the use of the app on first use” [Participant #17, registrar] and noted that the app should not be used in “isolation.” Another physician suggested the “addition of ethnicity in future” [Participant #6, consultant] iterations of the model as an important prognostic factor. Another physician suggested “linking trust-based guidelines for COVID-19 management” on the results page of the app, or “integrating the results into the patient’s electronic health records” [Participant #13, registrar].

Two physicians noted that it should be made clear that duration of symptoms is always recorded from the onset of first symptom by the app: “I think you should specify that the duration of symptoms is from the first symptom, as sometimes symptoms develop at different time points” [Participant #16, registrar]. Finally, being able to predict “intensive care requirements” [Participant #6, consultant] and “prolonged hospital stay” [Participant #4, consultant] were considered useful improvements to the algorithm.

#### User-Derived Concerns Regarding the ANN-Based COVID-19 Prognostication App

The principal concern expressed by users was the use of the predictions as an exclusive decision-making tool by, for example, making “management setting and treatment escalation decisions based solely on the results” [Participant #5, senior house officer] of the app.

I think a discussion may be required with ICU before deciding on ward-based care, and I’d worry if a high mortality prediction led to an automatic decision to not admit to ICU.

Participant #2, registrar

There were concerns that “generalizability would be difficult” [Participant #1, registrar] since the data are accrued from admissions to a single center: “Different patients in the UK will have different cohorts and so it should be generalized with caution” [Participant #8, senior house officer].

The model underlying this app was trained with patients during the first wave of COVID-19 in the United Kingdom. There were no established management guidelines or prognostic scoring system relating to this disease. Several physicians noted the importance of retraining the model with more recent data from patients with COVID-19 to reflect recent developments in the management of this condition: “The guidelines are changing, and so the data itself may change” [Participant #14, consultant]; therefore, “the application may not be calibrated to new waves, given newer treatments” [Participant #29, registrar]. The same physician indicated that “there is little concern if this is used as part of the big picture but shouldn’t be used in a binary sense” [Participant #29, registrar]. This sentiment was echoed by several other physicians who felt that “you have to be responsible and realize no predictive calculator is a substitute for clinical judgement- I don’t think anyone should be under the impression that a calculator can replace their judgement entirely” [Participant #7, registrar].

## Discussion

### Principal Findings

We tested the clinical utility of a responsive web-based app or GUI, which interfaces to an ANN to predict the outcomes of patients with COVID-19 at the bedside. All clinician-users were able to use the GUI with a mean time of 59.35 (SD 10.35) seconds to derive a mortality prediction. We found that clinician-users assigned a mean SUS score of 91.94 (SD 8.54), which corresponds to an adjective rating of “excellent.” Clinician-users found the app intuitive and easy to use, and the majority described its predictions as a useful adjunct to their clinical practice. The main concerns were related to the use of the app in isolation rather than in conjunction with other clinical parameters. However, most clinicians felt that the app could positively reinforce or validate their clinical decision-making. Effectiveness and efficiency measures indicated that the app could be used easily with little technical support or prior explanation with respect to system function. The app is therefore highly productive, while maintaining low costs and learnability times. No participant took longer than 2.2 minutes to successfully input all required patient data and retrieve a prediction.

Thematic framework analysis provided further insight into the implications of the use of this app. The identification of deteriorating patients with COVID-19 was a key concern for most physicians. From a clinical perspective, accurate risk stratification underpins hospital admission decisions, as well as appropriate ceilings of patient management. Furthermore, an understanding of risk allows physicians to better communicate prognoses to the patient and their relatives. Hence, a large majority of participants in this study felt that a scoring system can be a useful as an adjunct to their clinical workflow and could aid in communicating risk to patients and their families. However, most physicians agreed that the use of a predictive scoring system alone cannot surmount the decision-making by a clinician.

The spectrum of opinions regarding mortality risk predictions when faced with the same clinical scenario highlighted variations among clinicians. This emphasizes the potential role of an easy-to-use, widely accessible predictive system in minimizing biases such as experiential bias and the availability heuristic in prognostication.

### Strengths and Limitations

A strength of this study is that both usability assessments and a qualitative framework were used to evaluate the app, thereby providing a deeper insight into all aspects of its use and implications. In addition, multiple researchers analyzed the thematic framework data, ensuring consensus with regard to the results and their interpretation.

However, there are limitations to consider in this analysis. The study participants had high self-reported levels of expertise in using computers and smartphones. If this app were to be used in settings where users had limited experience in using clinical decision-making tools, it may impact usability, and subsequently affect results and result interpretation. Furthermore, the underlying algorithm is trained with patients from a single West London hospital site during the first wave of COVID-19 in the United Kingdom. The generalizability of its predictions is therefore reduced among other populations, and further studies need to evaluate the app in other health care settings.

### Comparison With Prior Studies and Future Prospects

Given that treatment for COVID-19 has progressed—for example, a recent study reported that dexamethasone reduces mortality in hospitalized patients with COVID-19 [25]—it is important to retrain or update the algorithm with new data to maximize the prognostic accuracy of the app. The adaptive nature of ANNs with their ease of retrainability, and the continued deposition of clinical big data for patients with COVID-19, implies that these latter limitations can be mitigated with future iterations.

The principal challenges in deploying AI technologies in a pandemic include the rapidly shifting clinical needs that the models need to address, and in translating these models to local environments [5]. While numerous recent studies have been using machine learning processes for aspects of COVID-19 clinical care in various settings [26-30], few use co-design, as we have in this study, to optimize the utility of the app among clinicians. Furthermore, beyond user interface and utility challenges lie ethical and legal issues that are inherent when smartphone apps are used as health care decision support systems [31]. The ethical aspects of integrating computerized decision support systems in to the management of infectious diseases remain unclear, but the importance in co-design with clinician-users early on in the preimplementation phase (as in this study) takes precedence to ensure that clinicians use them as part, rather than the entirety, of their overarching clinical assessment [32,33].

**Figure 3:**
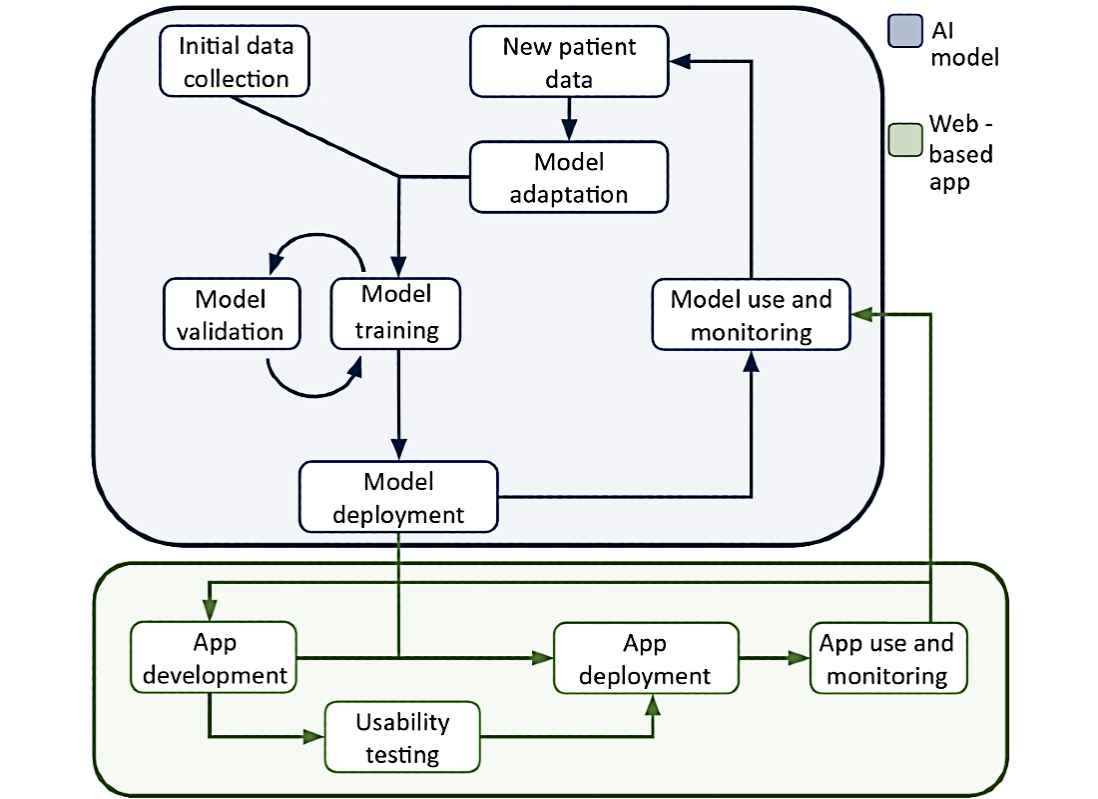
Proposed translational network for the artificial neural network and web-based app, including model training, validation, and adaptation, as well as app development, testing, and deployment. AI: artificial intelligence.

Based on our development of the ANN [4] and the clinical utility and feasibility assessment undertaken in this analysis, we propose an adaptive translational pathway for predictive systems for COVID-19 (Figure 3). This workflow recognizes the need for feedback mechanisms in the development and deployment of both the GUI and its underlying AI algorithm. As management strategies shift, new data must be incorporated through web-based learning or retraining of the algorithm to maintain accurate predictions. The new models then require further validation on test data sets to ensure reliability. In tandem, the application must be actively monitored for usability and security issues and updated as appropriate. Utilizing interconnected feedback mechanisms in this way can ensure that both the algorithm and the interface to it remain robust to changing trends in patient cohorts and the management of COVID-19.

**Figure 4:**
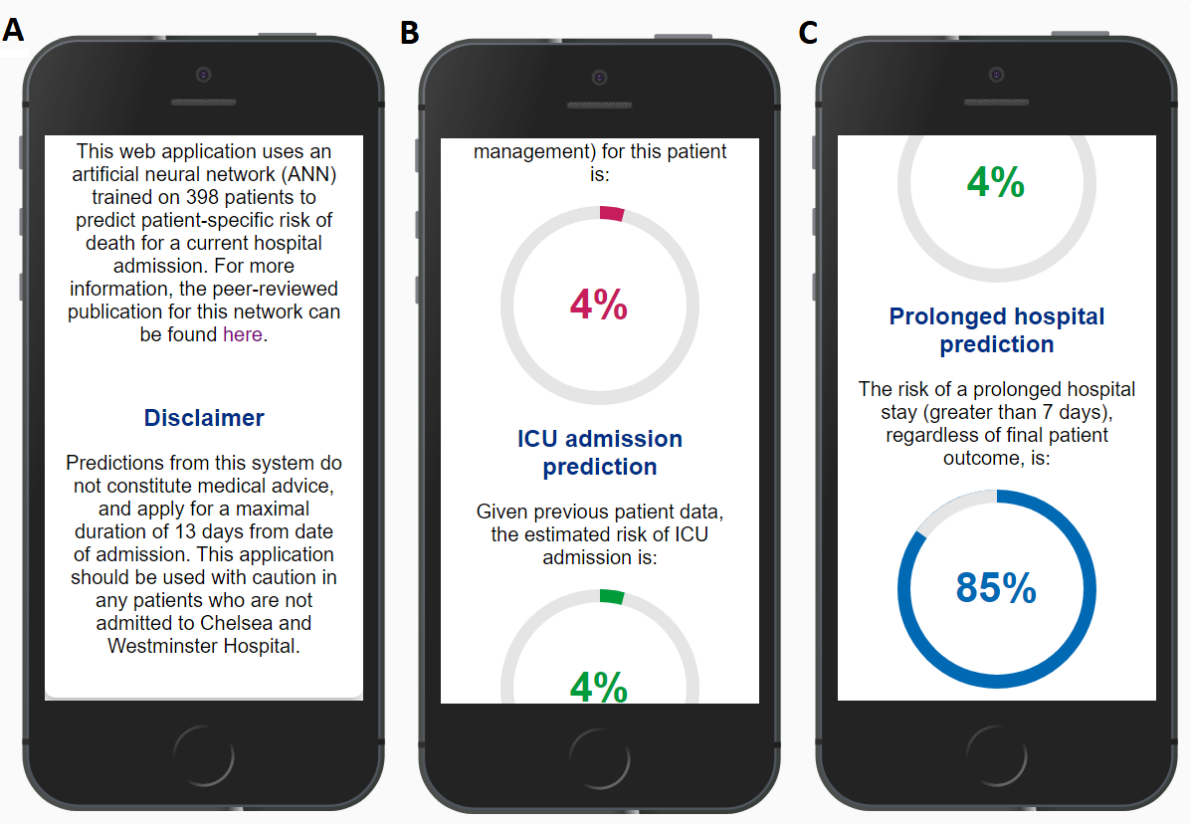
Screenshots of the matured artificial neural network–based COVID-19 prognostication app. (A) The introductory screen and an added disclaimer for use. (B) and (C) A portion of the results screen. Predictions regarding mortality, intensive care unit, and prolonged hospital stay are presented as human-readable percentages and are color-coded to reflect retraining of the underlying algorithm.

Following this framework, because of the usability assessment and thematic framework analysis, our current app was modified to include several of the suggested improvements. These included, but were not limited to, the addition of a disclaimer on the index page and retraining the algorithm to estimate mortality, probability of admission to an intensive care unit, and probability of a prolonged hospital stay (defined as a stay of at least 1 week). These changes are shown in Figure 4. Future improvements include model retraining from patient samples across multiple hospital sites, and the potential integration of the app to patient electronic health records to facilitate its use in the context of clinicians’ workflow, although the barriers to integration into electronic medical records are numerous [34].

### Conclusions

Developing, validating, and deploying AI technologies in health care is associated with a variety of challenges. In this single hospital study, we tested a responsive web-based app, which leverages an ANN to produce multiple outcome predictions for patients with COVID-19 without the need for laboratory parameters. It demonstrates potential utility among patients with an initial presentation of COVID-19 and for those without diagnostic capability in the community. The application is intuitive and requires minimal training for use. Clinicians interviewed in this study found that the system represents a useful adjunct to their daily clinical practice, and we propose a translational workflow for future predictive systems that leverage similar technologies. We demonstrate that both model and interface adaptation can be used to meet the developing needs of clinicians in the context of a pandemic.

## Abbreviations

AI: artificial intelligence
ANN: artificial neural network
GP: general practitioner
GUI: graphical user interface
HPRU: Health Protection Research Unit
ICU: intensive care unit
NIHR: National Institute for Health Research
SUS: system usability score
